# Unexpected diversity and a new species of *Epizoanthus* (Anthozoa, Hexacorallia) attached to eunicid worm tubes from the Pacific Ocean

**DOI:** 10.3897/zookeys.562.6181

**Published:** 2016-02-10

**Authors:** Hiroki Kise, James Davis Reimer

**Affiliations:** 1 Molecular Invertebrate Systematics and Ecology Laboratory, Graduate School of Engineering and Science, University of the Ryukyus, 1 Senbaru, Nishihara, Okinawa 903-0213, Japan; 2 Tropical Biosphere Research Center, University of the Ryukyus, 1 Senbaru, Nishihara, Okinawa 903-0213, Japan

**Keywords:** Eunicidae, cryptic species, mesophotic, molecular analyses, underwater cave, zoantharian

## Abstract

*Epizoanthus* species are generally found in association with other marine invertebrates such as hermit crabs and gastropods. Although *Epizoanthus* spp. are relatively common, there is limited information about their diversity and ecology due to their habitats or hosts, often being below the depths of SCUBA diving (>~50 m). In particular, the *Epizoanthus* fauna of the Indo-Pacific Ocean remains poorly understood. In this study, the diversity of *Epizoanthus* species associated with eunicid worm tubes from shallow waters in the Pacific Ocean we investigated using molecular analyses (mitochondrial cytochrome oxidase subunit 1 = COI, mitochondrial 16S ribosomal DNA = mt 16S-rDNA, nuclear internal transcribed spacer region of ribosomal DNA = ITS-rDNA) combined with morphological and ecological data. The combined data set leads us to describe two new species; *Epizoanthus inazuma* **sp. n.** and *Epizoanthus beriber* **sp. n.** Both new species are found in low-light environments: *Epizoanthus inazuma* **sp. n.** on mesophotic coral reef slopes and reef floors, or on the sides of overhangs; *Epizoanthus beriber* **sp. n.** has only been found in caves. Morphological characteristics of these two new species are very similar to *Epizoanthus illoricatus* Tischbierek, 1930 but the two new species are genetically distinct. Mesentery numbers and coloration of polyps may be useful diagnostic characteristics among eunicid-associated *Epizoanthus* species. These results demonstrate that there is high potential for other potentially undescribed zoantharian species, particularly in underwater cave habitats.

## Introduction

The order Zoantharia is currently separated into two suborders (Haddon and Shackleton 1891): Macrocnemina and Brachycnemina. The suborders are distinguished by differences in the fifth pair of mesenteries from the dorsal directive, which are complete in the suborder Macrocnemina and incomplete in the suborder Brachycnemina. The suborder Macrocnemina is currently composed of five families: Epizoanthidae, Hydrozoanthidae, Microzoanthidae, Nanozoanthidae, and Parazoanthidae. Most species of Macrocnemina to the exception of Microzoanthidae and Nanozoanthidae are often found in association with other marine invertebrates. The family Epizoanthidae can be distinguished from other macrocnemic zoantharians by the presence of a simple mesogloeal sphincter muscle.

The family Epizoanthidae consists of three genera: *Epizoanthus*, *Palaeozoanthus*, and *Thoracactis*. The genus *Palaeozoanthus* has not been found or examined in detail since its original description (Carlgren 1924), while *Thoracactis topsenti* Gravier, 1918 is the sole representative of its genus and is an epibiont on sponges at 800-1100 meters around the Cape Verde Islands (Gravier 1918). The type genus of Epizoanthidae, *Epizoanthus*, includes species that have epibiotic associations with hermit crabs (Muirhed et al. 1986; Ates 2003; Reimer et al. 2010a; Schejter and Mantelatto 2011), molluscs (Rees 1967), eunicid worms (Sinniger et al. 2005), or the stalks of glass sponges (hexactinellids) (Beaulieu 2001). *Epizoanthus* spp. have been reported worldwide, including from the northeast Atlantic (Muirhead et al. 1986), the Caribbean Sea (Duerden 1898), and both the eastern (Carlgren 1899; Philipp and Fautin 2009; Sinniger et al. 2009) and western Pacific (Haddon and Shackleton 1891; Reimer et al. 2010a).

Although *Epizoanthus* spp. are relatively common, little research has been conducted on the ecology and taxonomy of this genus (Ates 2003). Many *Epizoanthus* species are known from below the depth limits safe for SCUBA diving (>~50 m), making collection and observation difficult. *Epizoanthus* species are also often difficult to identify due to lack of external diagnostic characteristics, and data are often limited to polyp size, oral disk color, and tentacle count (Reimer et al. 2010a). It is often difficult to observe zoantharian internal morphology due to sand encrustation in their epithelial/endodermal tissue, making thin cross sections difficult without compromising histology (Reimer et al. 2010b). Molecular phylogenetic analyses have been used to overcome these issues and to help understand zoantharian diversity and taxonomy (e.g. Burnett 1997; Reimer et al. 2006; Sinniger et al. 2008; Fujii and Reimer 2011). For example, *Epizoanthus* species diversity in Japan has been preliminarily investigated by using molecular methods and potentially undescribed species were found (Sinniger et al. 2009; Reimer et al. 2010a). Thus, molecular methods are an effective tool to help clarify the taxonomy and diversity of *Epizoanthus* species.

There are several described *Epizoanthus* species which are free-living, carcinoecium-forming, or epizoic on gastropods or glass sponges from the Pacific Ocean, such as *Epizoanthus paguriphilus* Verrill, 1883 from the East China Sea; *Epizoanthus stellaris* Hertwig, 1888 from the Philippines; *Epizoanthus patagonichus* Carlgren, 1899 from Chile; *Epizoanthus indicus* (Lwowsky, 1913) from the East China Sea; *Epizoanthus illoricatus* Tischbierek, 1930 from Manila; *Epizoanthus ramosus* Carlgren, 1936 from the East China Sea; *Epizoanthus scotinus* Wood, 1957 from the Pacific Northwest; *Epizoanthus sabulosus* Cutress, 1971 from Australia; *Epizoanthus giveni* Philipp & Fautin, 2009 from California, and *Epizoanthus fiordicus* Sinniger & Haussermann, 2009 from Chile. For *Epizoanthus* spp. attached to zig-zag shaped eunicid worm tubes, all have been identified as *Epizoanthus illoricatus* since the species’ original description. Eunicid worms are distributed in marine benthic environments around the world and are especially common in shallow tropical waters (Fauchald 1992). The family Eunicidae is currently composed of eight valid genera and ~330 species (Zanol et al. 2013), some of which are known to have associations with various marine invertebrates such as cnidarians, sponges and mollusks (Martin and Britayev 1998; Neves and Omena 2003). Recently, Reimer et al. (2014) investigated the diversity of zoantharians in the central Indo-Pacific and suggested that there are may be at least two species within *Epizoanthus illoricatus*. However, no taxonomic conclusions were reached in this study.

In the current study the diversity of *Epizoanthus* species attached to eunicid worm tubes we investigated via molecular phylogenetic analyses utilizing three DNA markers; nuclear internal transcribed spacer of ribosomal DNA (ITS-rDNA), mitochondrial 16S ribosomal DNA (mt 16S-rDNA), and cytochrome oxidase subunit I(COI), and nuclear internal transcribed spacer of ribosomal DNA (ITS-rDNA). We then combined molecular results with morphological data (polyp dimensions, polyp arrangement and density within colonies, external colony and oral disk color, cnidae analyses, mesenterial patterns and numbers). The combined results of this research indicated the presence of two phylogenetically distinct and previously undescribed species of *Epizoanthus* associated with eunicid worm tubes in the Pacific Ocean, which we formally describe herein.

## Materials and methods

### Specimen collection

**Figure 1.**
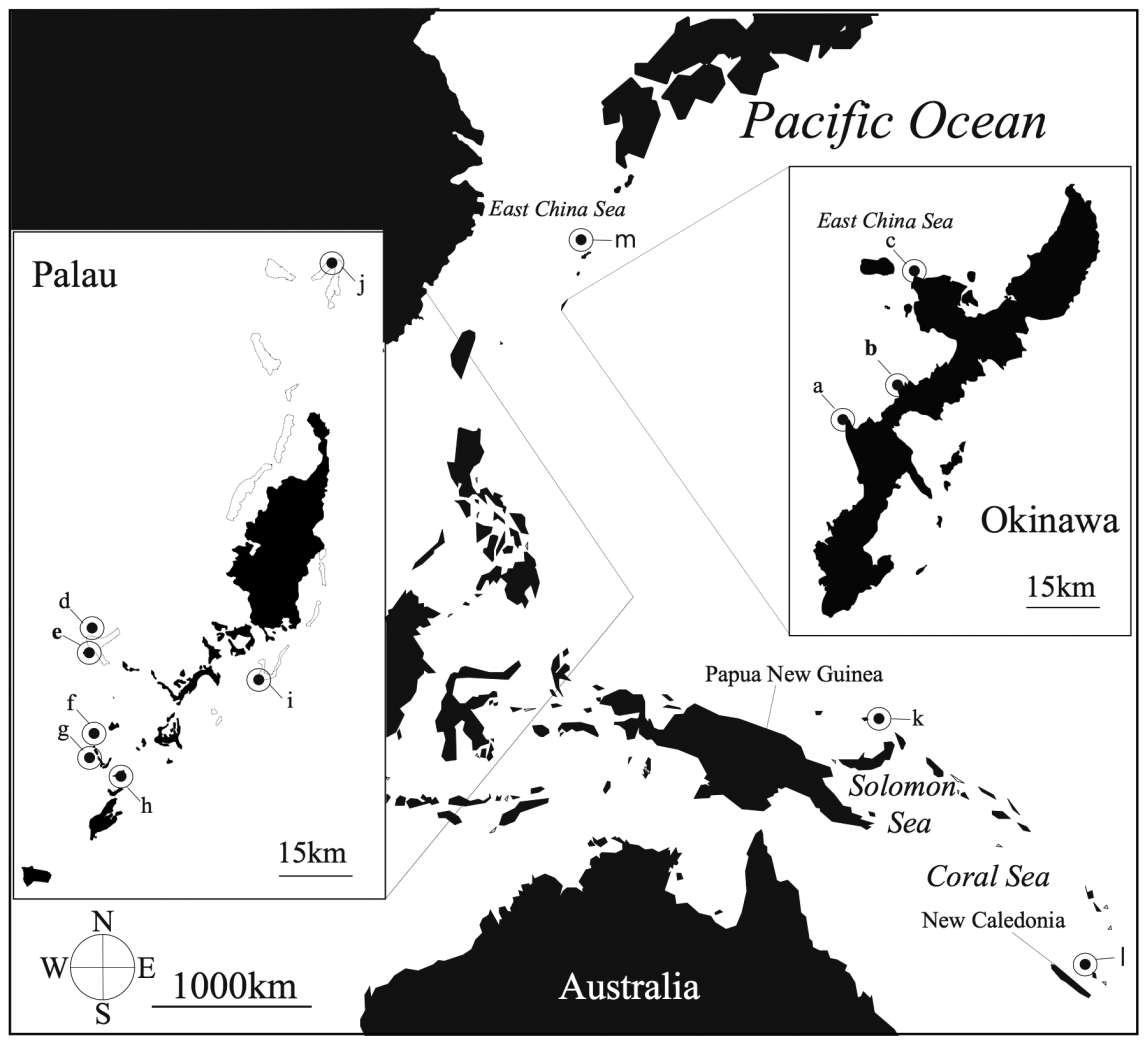
Sampling location in the Pacific Ocean of specimens used in this study. Location of specimens collected in this study represented by closed symbols. **a** Cape Zanpa **b** Cape Manzamo **c** Bise **d** Siaes Corner **e** Siaes Tunnel **f** Blue Hole **g** Blue Corner **h** Turtle Cove **i** Short Drop-off **j** Ngeruangel **k** Mascot Channel **l** Loyalty Islands **m** Kodakara Islands. Location in bold indicate type localities as follows: **b** (Cape Manzamo, Onna, Okinawa, Japan) = *Epizoanthus inazuma* sp. n. **e** (Turtle Cove, Palau) = *Epizoanthus beriber* sp. n.

*Epizoanthus* specimens were collected from three localities in Okinawa, Japan, seven localities in Palau, and one location each in New Caledonia and Papua New Guinea (Figure 1). In total 70 specimens were collected, of which 69 specimens were collected by SCUBA at 10 to 40 m depth, with one additional specimen collected using the Japan Agency for Marine-Earth Science and Technology (JAMSTEC)’s ROV Hyper-Dolphin from 114 m during a research cruise in southern Japan in 2012. Collected specimens were preserved in 70–99.5% ethanol for molecular analyses and/or fixed in 5–10% seawater formalin and then later preserved in 70% ethanol for morphological analyses.

### Morphological analyses

The lengths and diameters of individual polyps, tentacle lengths and numbers, color of polyps, and diameters of oral disks were measured using in situ images or a dissecting microscope. Additionally, polyp densities of colonies attached to identically sized eunicid worm tubes (9 cm in length) were calculated using a counter under a dissecting microscope. For internal morphological analyses, some specimens’ polyps were cut into 7 µm cross-sections using a microtome after paraffin embedding following Reimer et al. (2010b), and these sections were subsequently stained with hematoxylin and eosin. Specimens to examine were selected from each phylogenetic clade (n = 3/clade) recovered in the molecular analyses.

### Cnidae

Cnidae classification basically followed England (1991) and Ryland and Lancaster (2004). However, Schmidt (1974), Hidaka et al. (1987), Hidaka (1992), Fujii and Reimer (2011), and Montenegro et al. (2015) have referred to basitrichs and microbasic b-mastigophores as the same type of nematocysts and therefore in this study, these two types were pooled together. We used a Nikon Eclipse80i stereomicroscope (Nikon, Tokyo) to count and examine undischarged cnidae, which were measured using ImageJ software (National Institute of Health, Bethesda, Maryland, Nikon Eclipse80i, Nikon, Tokyo). Specimens to be examined were selected from each phylogenetic clade recovered from the molecular analyses.

### DNA extraction, PCR amplification and sequence

DNA was extracted from tissue preserved in 99.5% ethanol by following a guanidine extraction protocol (Sinniger et al. 2010) or using a spin-column DNEasy Blood and Tissue Extraction kit (Qiagen, Tokyo). PCR amplification using Hot Star Taq Plus Master Mix Kit (Qiagen, Tokyo) was performed for each of ITS-rDNA (nuclear internal transcribed spacer region of ribosomal DNA), mt 16S-rDNA (mitochondrial 16S ribosomal DNA), and COI (cytochrome oxidase subunit I). The ITS-rDNA region was amplified using the specific primer set ITSf (5’-CTA GTA AGC GCG AGT CAT CAG C-3’) and ITSr (5’-GGT AGC CTT GCC TGA TCT GA-3’) (Swain 2009). mt 16S-rDNA was amplified using the universal primer 16Sar (5’-CGC CTG TTT ATC AAA AAC AT-3’) (Palumbi et al. 1996) and the specific primer 16SBmoH (5’-CGA ACA GCC AAC CCT TGG3’) (Sinniger et al. 2005). The COI gene was amplified using the universal primer set LCO1490 (5’-GGT CAA CAA ATC ATA AAG ATA TTG G-3’) and HCO2198 (5’-TAA ACT TCA GGG TGA CCA AAA AAT CA-3’) (Folmer et al. 1994). All DNA markers were amplified following the thermal-cycle conditions described in Fujii and Reimer (2011). PCR products were checked using 1.0% agarose gel electrophoresis. The positive PCR products were cleaned using shrimp alkaline phosphatase (SAP) and Exonuclease I (Takara Bio Inc., Shiga, Japan), and then sequenced by Fasmac (Kanagawa, Japan).

### Phylogenetic analyses

Obtained DNA sequences were initially checked using the Basic Local Alignment Search Tool (BLAST, National Center for Biotechnology Information). Obtained nucleotide sequences for the COI gene, mt 16S-rDNA and ITS-rDNA were aligned by CLUSTAL W ver. 1.83 (Thompson et al. 1994) on default settings supplied by Bioedit ver. 7.0.9.0. (http://www.mbio.ncsu.edu/Bioedit/page2.html). The alignments were inspected by eye and manually edited in Bioedit. Sequences belonging to the family Hydrozoanthidae were used as outgroups. In this manner three aligned datasets were generated. All sequence datasets are available upon request from the corresponding author.

For the phylogenetic analyses of ITS-rDNA, mt 16S-rDNA and COI, the same methods were independently applied. The neighbor-joining (NJ) method (Saitou and Nei 1987) was performed using MEGA6 (Tamura et al. 2013), with 1000 replicates of bootstrapping. Maximum-likelihood (ML) analyses were performed using PhyML online (Guindon and Gascuel 2003). PhyML was performed using an input tree generated by BIONJ with the general time-reversible model (Rodriguez et al. 1990) of nucleotide substitution incorporating invariable sites and a discrete gamma distribution (eight categories) (GTR+I+C). The proportion of invariable sites, a discrete gamma distribution, and base frequencies of the model were estimated from the dataset. PhyML bootstrap trees (1000 replicates) were constructed using the same parameters as the individual ML trees. Bayesian trees were constructed in Mr Bayes 3.1.2 (Ronquist and Huelsenbesk 2003) under the GTR + I + I- model. One cold and three heated Markov chain Monte Carlo (MCMC) chains with temperature of 0.2 were run for 1,500,000 generations, subsampling frequency of 200 and a burn in length of 700,000 for all alignments.

## Results

### Systematics

Phylum Cnidaria Hatschek, 1888

Class Anthozoa Ehrenberg, 1831

Subclass Hexacorallia Haeckel, 1896

Order Zoantharia Gray, 1832

Suborder Macrocnemina Haddon & Shackleton, 1891

Family Epizoanthidae Delage & Hérouard, 1901

Genus *Epizoanthus* Gray, 1867

#### Epizoanthus

Taxon classificationAnimaliaZoanthariaEpizoanthidae

Gray, 1867

##### Type species.

*Epizoanthus papillosus* Johnston, 1842.

##### Synonym.

*Epizoanthus incrustatus* (Dueben & Koren, 1847) (ICZN 1991: case 2750).

##### Remark.

Herein, we choose to use the ordinal name Zoantharia Gray, 1832 as in the World Register of Marine Species (Hoeksema and Reimer, 2015). Although Zoantharia Gray, 1832, has identical spelling with the supraordinal name Zoantharia de Blainville, 1830, the latter name has fallen from common use—Hexacorallia Haeckel, 1896, being favoured.

#### Epizoanthus inazuma sp. n.

Taxon classificationAnimaliaZoanthariaEpizoanthidae

http://zoobank.org/0B91DB0E-A5AC-41CB-B78F-C8E7B8D44C2A

##### Material examined.

Holotype. NSMT-Co1574 (MISE-HK54), 26°30'18.3"N, 127°51'02.3"E, Cape Manzamo, Onna Village, Okinawa, Japan, depth 24 m, collected by Hiroki Kise, July 21, 2014, divided in two pieces, one portion fixed in 99.5% EtOH and the other in 5–10% saltwater formalin, deposited in National Museum of Nature and Science, Tokyo, Japan. Paratype 1. RMNH 42100 (MISE-HK9) 26°30'18.3"N, 127°51'02.3"E, Cape Manzamo, Onna Village, Okinawa, Japan, depth 25 m, collected by James D. Reimer, October 21, 2008, fixed in 99.5% EtOH, deposited in Naturalis Biodiversity Center, Leiden, The Netherlands. Paratype 2. USNM 1296757 (MISE-HK66) 26°26'26.5"N, 127°42'43.7"E Cape Zanpa, Yomitan Town, Okinawa, Japan, depth 34 m, collected by Hiroki Kise, August 5, 2014, fixed in 99.5% EtOH, deposited in Smithsonian Institution National Museum of Natural History, Washington, D.C., USA. Other material. MISE-HK43 26°30'18.3"N, 127°51'02.3"E, Cape Manzamo, Onna Village, Okinawa, Japan, depth 30 m, collected by Hiroki Kise, April 5, 2014, fixed in 99.5% EtOH.

##### Description of holotype.

Colony of approximately 140 polyps connected by thin, under-developed coenenchyme on eunicid worm tubes. The tubes are made of a chitin-like substance. Polyps approximately 0.7 to 1.2 mm high (=length) from coenenchyme, and 1.0 to 1.65 mm in diameter. Polyps were attached from base to proximal extremity of zig-zag shaped tubes of eunicid worms, and attached to not only bent sections but also to other locations. Polyp external coloration black, oral disk light brown to brown, lighter nearer the oral opening and darker around oral disk edges. Polyps encrusted with sand and silica particles in their coenenchyme and ectodermal tissue; with few sand particles in the mesoglea.

##### Diagnosis.

*Morphology*. Polyps connected by thin, under-developed coenenchyme on eunicid worms belonging to family Eunicidae. Maximum diameter of polyps approximately 4 mm, maximum height approximately 5 mm in situ. *Epizoanthus inazuma* sp. n. has 20-22 tentacles that are cylindrical and either as long or longer in comparison to oral disk diameter.

**Figure 2.**
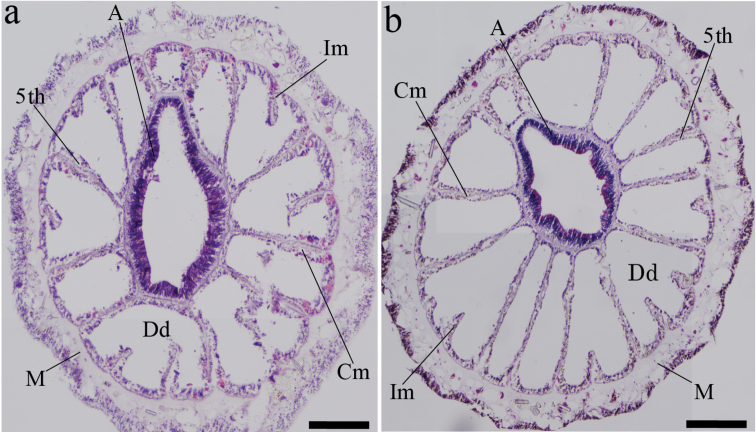
Cross-sections of *Epizoanthus illoricatus* and *Epizoanthus inazuma* sp. n. **a** *Epizoanthus illoricatus*; 6^th^ mesentery is incomplete from dorsal directive **b** *Epizoanthus inazuma* sp. n. 6^th^ mesentery is complete from dorsal directive. **Dd** dorsal directive **A** actinopharnx **Im** incomplete mesentery **Cm** complete mesentery **M** mesoglea; 5^th^, 5^th^ mesentery from dorsal directive. Scale bars: 200 μm.

*Internal anatomy*. While the 5th mesentery from dorsal directive is obviously a complete mesentery (macrocnemic arrangement), the 6th mesentery is also a complete mesentery (Figure 2b). Azooxanthellate. Mesogleal thickness approximately 75 µm.

**Figure 3.**
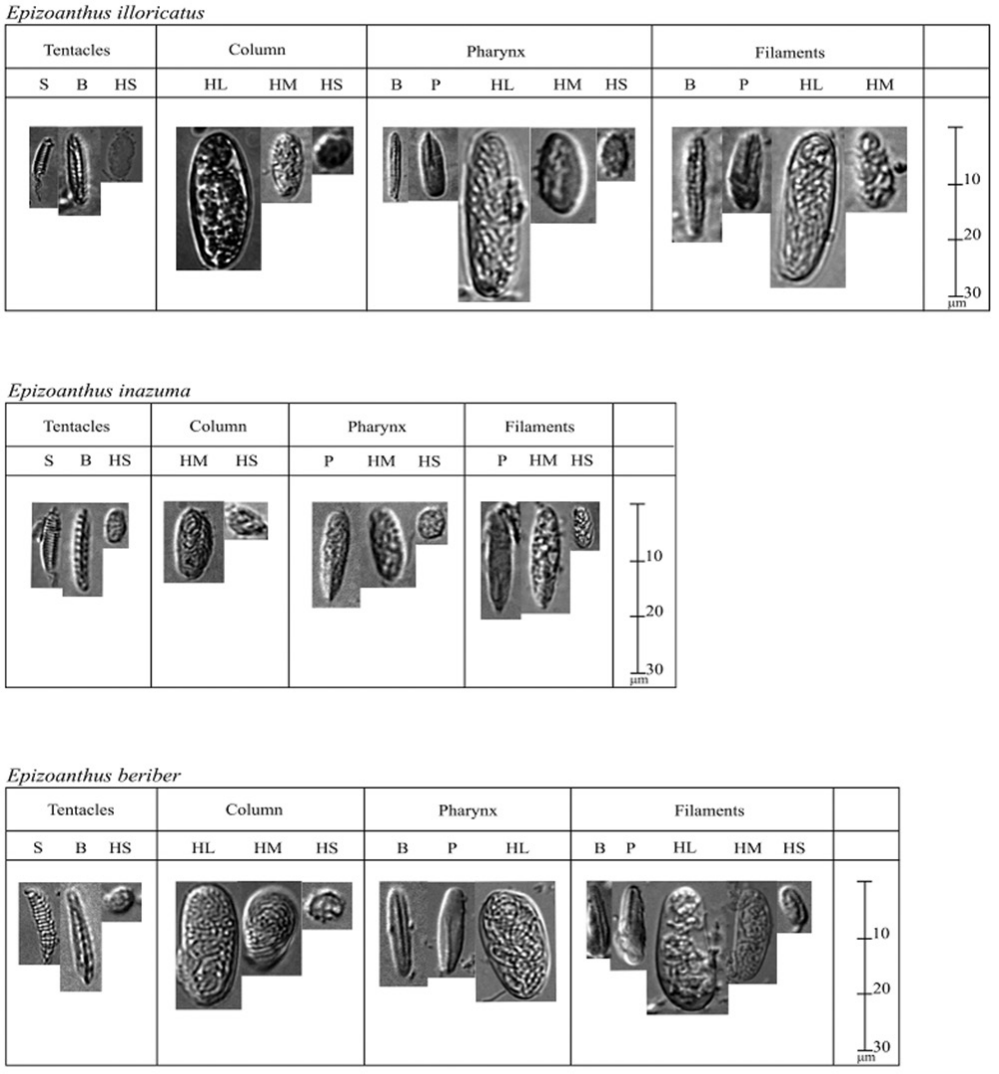
Cnidae in tentacles, column, pharynx, filaments of *Epizoanthus illoricatus*, *Epizoanthus inazuma* sp. n. and *Epizoanthus beriber* sp. n. respectively. **HL** holotrichs large **HM** holotrichs medium **HS** holotrichs small **B** basitrichs **pM** microbasic p-mastigophores **S** spirocysts.

*Cnidae*. Holotrichs, basitrichs, microbasic p-mastigophores, spirocysts (see Table 1 into this paper, Figure 3).

**Table 1.** Cnidae types and sizes of *Epizoanthus inazuma* sp. n., *Epizoanthus beriber* sp. n. and *Epizoanthus illoricatus*. Frequency: relative abundance of cnidae type in decreasing order; numerous, common, occasional, rare (N = number of specimens found/total specimens examined).”

	*Epizoanthus inazuma* sp. n.		*Epizoanthus beriber* sp. n.		*Epizoanthus illoricatus*	
	Length × Width (µm)	Frequency	Length × Width (µm)	Frequency	Length × Width (µm)	Frequency
Tentacles						
Spirocysts	8–20 × 1–4	Numerous (3/3)	8–21 × 2–5	Numerous (3/3)	9–20 × 2–4	Numerous (3/3)
Bastrichs	6–19× 1–5	Common (3/3)	9–18 × 2–4	Common (3/3)	10–23 × 2–5	Common (3/3)
Holotrichs small	4–9 × 2–4	Occasional (2/3)	5–7 × 2–4	Rare (1/3)	5–9 × 2–5	Occasional (2/3)
Column						
Holotrichs small	5–8 × 3–4	Common (3/3)	5 × 4	Rare (1/3)	5–8 × 2–4	Occasional (1/3)
Holotrichs medium	10–17 × 4–9	Numerous (3/3)	11–19 × 4–9	Numerous (3/3)	10–19 × 3–9	Numerous (3/3)
Holotrichs large	-	-	20–23 × 7–11	Occasional (3/3)	24–32 × 10–15	Occasional (1/3)
Actinopharnx						
P-mastigophores	11–20 × 3–5	Common (3/3)	17–25 × 4–6	Common (3/3)	12–19 × 3–5	Occasional (2/2)^a^
Bastrichs	-	-	14–19 × 2–4	Occasional (2/3)	9–20 × 1–4	Occasional (2/2)^a^
Holotrichs small	4–9× 1–6	Common (3/3)	-	-	6–9 × 2–4	Occasional (1/2)^a^
Holotrichs medium	10–19 × 4–6	Occasional (3/3)	-	-	11–16 × 5–8	Common (2/2)^a^
Holotrichs large	-	-	20–25 × 8–12	Occasional (2/3)	30 × 8	Rare (1/2)^a^
Mesenteries filaments						
Bastrichs	-	-	16–20 × 3–4	Occasional (2/3)	16–26 × 2–4	Occasional (1/3)
P-mastigophres	15–22 × 3–6	Common (3/3)	15–21 × 4–7	Common (3/3)	7–21 × 3–6	Common (3/3)
Holotrichs small	5–9 × 3–5	Common (3/3)	8 × 3	Rare (1/3)	-	-
Holotrichs medium	10–20 × 3–7	Common (3/3)	10–17 × 3–6	Occasional (3/3)	10–18 × 3–7	Common (3/3)
Holotrichs large	-	-	23–30 × 7–13	Occasional (2/3)	25–34 × 5–9	Occasional (1/3)

a Tissue of actinopharynx could be obtained from only two specimens due to specimen condition.

##### Etymology.

*Epizoanthus inazuma* sp. n. is named after the Japanese word ‘inazuma’ meaning ‘lightning’, as colonies of this species are attached to eunicid worm tubes, and the worm tube shape resembles a classic lightning-bolt shape. *Common Japanese name*. ‘Inazuma-yadori-sunaginchaku’ (new Japanese name).

##### Distribution and habitat.

*Epizoanthus inazuma* sp. n. is found in low-light environments such as on mesophotic coral reef slopes and reef floors, or on the sides of overhangs. Specimens were collected from 10 to 40 m depth.

*Epizoanthus inazuma* sp. n. is currently known only from Okinawa (Figure 1). However, it may be distributed in other locations in the Pacific Ocean, as it is likely this species has been confused with *Epizoanthus illoricatus* and/or *Epizoanthus beriber* sp. n. in the past due to their similar external morphology. *Epizoanthus illoricatus* has been found in many areas of the western Pacific Ocean such as in New Caledonia (Sinniger 2006; Sinniger et al. 2009), the Yellow Sea, China (Pei 1999), Papua New Guinea (BW Hoeksema, pers. comm.), Australia (Lindsay et al. 2012), Taiwan (Reimer et al. 2013), and Palau (Reimer et al. 2014), and *Epizoanthus inazuma* sp. n. may be similarly distributed.

##### Remarks.

**Figure 4.**
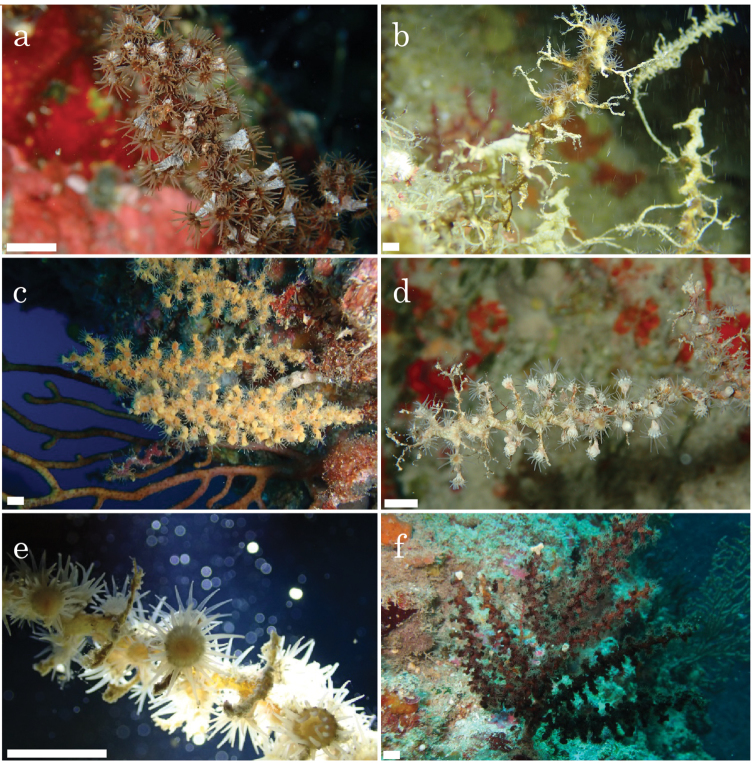
In situ images of *Epizoanthus illoricatus*, *Epizoanthus inazuma* sp. n. and *Epizoanthus beriber* sp. n. **a** *Epizoanthus illoricatus*; with highly developed coenenchyme and high density of polyps. Image taken on September 12, 2014, at Siaes Tunnel, Palau. Specimen number HK67. Image taken by J. D. Reimer **b** *Epizoanthus illoricatus*; with poorly developed coenenchyme and low density of polyps. Image taken on July 19, 2014, at Cape Manzamo, Okinawa, Japan. Specimen number HK53 **c** *Epizoanthus illoricatus*; yellow colored colonies. Image taken on November 21, 2015, at Cape Manzamo, Okinawa, Japan. Specimen number HK100 **d** *Epizoanthus beriber* sp. n.; with low density polyps. Image taken on May 6, 2015, at Turtle Cove, Palau. Specimen number HK129 (holotype) **e** *Epizoanthus beriber* sp. n.; open polyps. Image taken on April 28, 2015, at Siaes Tunnel, Palau. Specimen number HK113 **f** *Epizoanthus inazuma* sp. n.; black colored colony. Image taken on April 5, 2014, at Cape Manzamo, Okinawa, Japan. Specimen number HK54 (holotype). All images excepting specimen number HK67 taken by H. Kise. Scale bars: 3 cm.

*Epizoanthus inazuma* sp. n., *Epizoanthus beriber*, and *Epizoanthus illoricatus* can be distinguished from most other *Epizoanthus* species by their specific substrate (eunicid worm tubes) in the Pacific Ocean. *Acrozoanthus australiae* (family Zoanthidae) is also associated with eunicid worm tubes, but *Epizoanthus inazuma* sp. n. can be distinguished from *Acrozoanthus australiae* due to its mesenterial arrangement (the family Zoanthidae is within the suborder Brachycnemina), as well as by many obvious external features such as coloration, polyp size, and by being azooxanthellate (*Acrozoanthus australiae* is zooxanthellate). *Epizoanthus inazuma* sp. n. is very similar to *Epizoanthus illoricatus* (Figure 4a, b, c, f), but can be distinguished by differing mesenterial arrangement (6th mesentery is complete as opposed to 6th mesentery being incomplete in *Epizoanthus illoricatus*) (Figure 2). *Epizoanthus inazuma* sp. n. has different coloration than *Epizoanthus beriber* sp. n., which is pale white. *Epizoanthus inazuma* sp. n. and *Epizoanthus illoricatus* can have the same external coloration (black), but the cnidomes of these two species are different; *Epizoanthus illoricatus* has large holotrichs in the column, pharynx and mesenterial filaments, while *Epizoanthus inazuma* sp. n. does not have any large holotrichs in the column, pharynx, or mesenterial filaments. As well, there are also differences in sizes of some nematocyst types of these two species (e.g. bastrichs in the pharynx or mesenterial filaments). The cnidome composition of *Epizoanthus inazuma* sp. n. is different from *Epizoanthus beriber* sp. n. and *Epizoanthus illoricatus*, and *Epizoanthus beriber*’s sp. n. cnidome is similar to that of *Epizoanthus illoricatus* (see Table 1; Figure 3).

All Indo-Pacific *Epizoanthus* species that are obligate epibionts on eunicid worm tubes until now have been identified as *Epizoanthus illoricatus*, which was originally described from Manila, the Philippines. The type specimens of *Epizoanthus illoricatus* were likely lost during World War II when the Zoologische Staatssammlung Museum in München was burned down. Additionally, no specific type locality was given except ‘Manila’ in the original description and Manila is now a very altered environment compared to 1930. Therefore, it is difficult to find and identify *Epizoanthus illoricatus*’ exact type locality. However, *Epizoanthus illoricatus* can be clearly separated from *Epizoanthus inazuma* sp. n. and *Epizoanthus beriber* sp. n. by both morphological and molecular data.

#### Epizoanthus beriber sp. n.

Taxon classificationAnimaliaZoanthariaEpizoanthidae

http://zoobank.org/7F0A1F6F-4922-4C2C-AF62-33948394AC97

##### Material examined.

Holotype. NSMT-Co1575 (MISE-HK129), 7°5'01.0"N, 134°15'80.0"E, Turtle Cove, Palau, depth 20 m, collected by Hiroki Kise, May 6, 2015, divided in two pieces, one portion fixed in 99.5% EtOH and the other in 5–10% saltwater formalin, deposited in National Museum of Nature and Science, Tokyo, Japan. Paratype 1. RMNH 42101 (MISE-HK126), 7°8'29.4"N, 134°13'23.3"E, Blue Hole, Palau, depth 36 m, collected by Hiroki Kise, May 5, 2015, divided in two pieces, one portion fixed in 99.5% EtOH and other in 5–10% saltwater formalin, deposited in Naturalis Biodiversity Center, Leiden, The Netherlands. Paratype 2. USNM 1296758, USNM 1296759 (MISE-HK113), 7°18'54.8"N, 134°13'13.3"E, Siaes Tunnel, Palau, depth 30 m, collected by Hiroki Kise, April 28, 2015, divided in two pieces, one portion fixed in 99.5% EtOH and other in 5–10% saltwater formalin, deposited in Smithsonian Institution National Museum of Natural History, Washington, D.C., USA. Other material. MISE-HK112, 7°18'54.8"N, 134°13'13.3"E, Siaes Tunnel, Palau, depth 37 m, collected by Hiroki Kise, April 28, 2015, divided in two pieces and fixed in 99.5% EtOH and 5–10% saltwater formalin, respectively; MISE-HK116, 7°18'54.8"N, 134°13'13.3"E, Siaes Tunnel, Palau, depth unknown, collected by Hiroki Kise, April 28, 2015, divided in two pieces and fixed in 99.5% EtOH and 5–10% saltwater formalin, respectively; MISE-HK117, 7°18'54.8"N, 134°13'13.3"E, Siaes Tunnel, Palau, depth unknown, collected by Hiroki Kise, April 28, 2015, fixed in 99.5% EtOH; MISE-HK118, 7°18'54.8"N, 134°13'13.3"E, Siaes Tunnel, Palau, depth unknown, collected by Hiroki Kise, April 28, 2015, fixed in 99.5% EtOH; MISE-HK119, 7°18'54.8"N, 134°13'13.3"E, Siaes Tunnel, Palau, depth 19 m, collected by Hiroki Kise, April 28, 2015, fixed in 99.5%; MISE-HK120, 7°18'54.8"N, 134°13'13.3"E, Siaes Tunnel, Palau, depth unknown, collected by Hiroki Kise, April 28, 2015, fixed in 99.5% EtOH; MISE-HK124, 8°19'00.0"N, 134°63'00.0"E, Negruangel, Palau, depth 27 m, collected by Hiroki Kise, April 29, 2015, fixed in 99.5% EtOH; MISE-HK125, 7°8'29.4"N, 134°13'23.3"E, Blue Hole, Palau, depth 32 m, collected by Hiroki Kise, May 5, 2015, divided in two pieces and fixed in 99.5% EtOH and 5–10% saltwater formalin, respectively; MISE-HK127 7°8'29.4"N, 134°13'23.3"E, Blue Hole, Palau, depth 36 m, collected by Hiroki Kise, May 5, 2015, fixed in 99.5% EtOH; HK128 7°8'12.3"N, 134°13'16.5"E, Blue Corner, Palau, depth 29 m, collected by Hiroki Kise, May 5, 2015, fixed in 99.5% EtOH.

##### Description of holotype.

Colony of approximately 75 polyps connected by moderately developed coenenchyme on eunicid worm tubes. Polyps were attached to from base to proximal extremity of zig-zag shaped tubes of eunicid worms, and attached to not only bent sections but also to other locations. Polyps approximately 1.4 to 1.9 mm high from coenenchyme, and 0.7-1.0 mm in diameter. Azooxanthellate. Polyp external coloration is white, oral disk solid in color, ranging from light brown to brown (Figure 4d). Tentacles are transparent and approximately 20-22 in number.

##### Diagnosis.

*Morphology*. Polyps connected by moderately developed coenenchyme on eunicid worm tubes belonging to the genus *Eunice*, as are *Epizoanthus illoricatus* and *Epizoanthus inazuma* sp. n. Polyps are either circular cones or cylindrical, and approximately 0.5 to 2.1 mm high from coenenchyme (=length) and 1.1 to 2.1 mm diameter (in 5–10% seawater formalin). Maximum diameter of polyps is approximately 3 mm, maximum height approximately 5 mm in situ. Polyps have 20-22 tentacles that are longer than oral disk diameter. In addition, polyp external color is white while oral disk is light brown to brown.

*Internal anatomy*. Mesogleal thickness approximately 80 µm. We could not obtain cross-sections or images to observe mesentery arrangement due to heavy sand encrustation.

*Cnidae*. Holotrichs, basitrichs, microbasic p-mastigophores, spirocysts (see Table 1, Figure 3).

##### Etymology.

*Epizoanthus beriber* sp. n. is named after the legendary Beriber of Palauan folklore, who lived in a cave at Oikuul in Airai State, as this species has been found only in caves. *Common Japanese name*. ‘Ziguzagu-yadori-sunaginchaku’ (new Japanese name).

##### Distribution and habitat.

*Epizoanthus beriber* sp. n. is found only on the floor or sides of caves, and always in association with eunicid worm tubes (Figure 4d, e). Specimens were collected from 20-40 m in this study. *Epizoanthus beriber* sp. n. is known from Palau and Papua New Guinea (Figure 1). However, it may be distributed around the Pacific Ocean as we have speculated for *Epizoanthus inazuma* sp. n.

##### Remarks.

*Epizoanthus beriber* sp. n. can be distinguished from *Epizoanthus illoricatus* and *Epizoanthus inazuma* sp. n. by habitat and coloration. *Epizoanthus beriber* sp. n. was found only in caves while *Epizoanthus inazuma* sp. n. and *Epizoanthus illoricatus* were found on reef slopes or flat reef floors. *Epizoanthus beriber* sp. n. has white colonial polyps with a moderately developed coenenchyme (Figure 4d, e) while *Epizoanthus inazuma* sp. n. has black colonial polyps with a well-developed coenenchyme and *Epizoanthus illoricatus* has gray, yellow or black colonial polyps with a either poorly developed or well-developed coenenchyme (Figure 4a–c, f).

The holotype of *Epizoanthus illoricatus* was presumably collected by dredging or net as there was no SCUBA in the 1930s; and it can be inferred that the holotype of *Epizoanthus illoricatus* lived in a location where it could be collected by such a method, such as on a reef flat or reef slope. *Epizoanthus inazuma* sp. n. is also found in such areas. However, *Epizoanthus beriber* sp. n. is only known from underwater caves that cannot be easily accessed from the surface.

### Phylogenetic analyses

**Figure 5.**
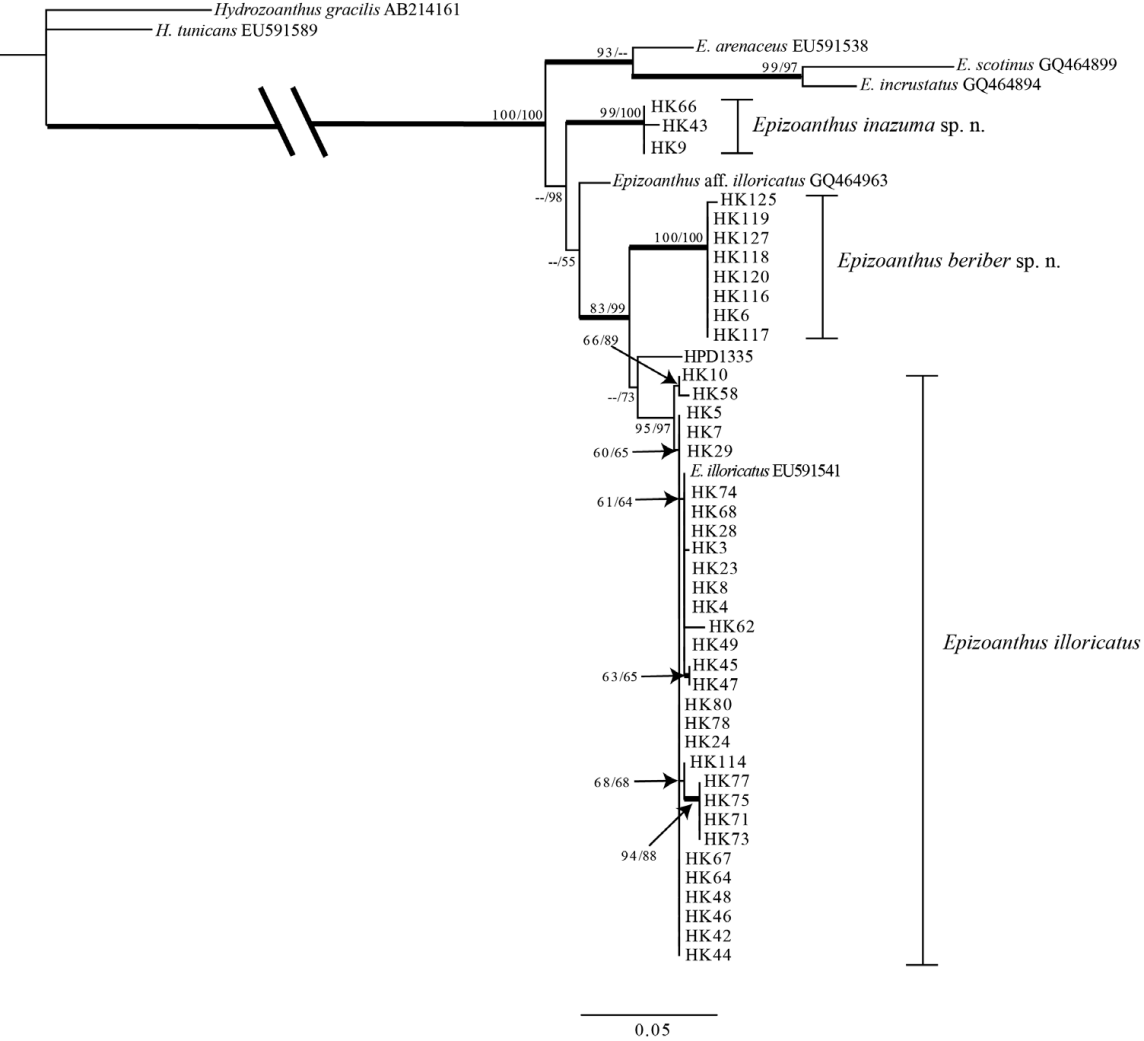
Maximum likelihood (ML) tree based on internal transcribed spacer region of ribosomal DNA sequence. Numbers on nodes represent ML and neighbor-joining (NJ) bootstrap values (> 50% are shown). Bold branches indicate high supports of Bayesian posterior probabilities (> 0.95). Sequences obtained from GenBank are shown with accession numbers.

**Figure 6.**
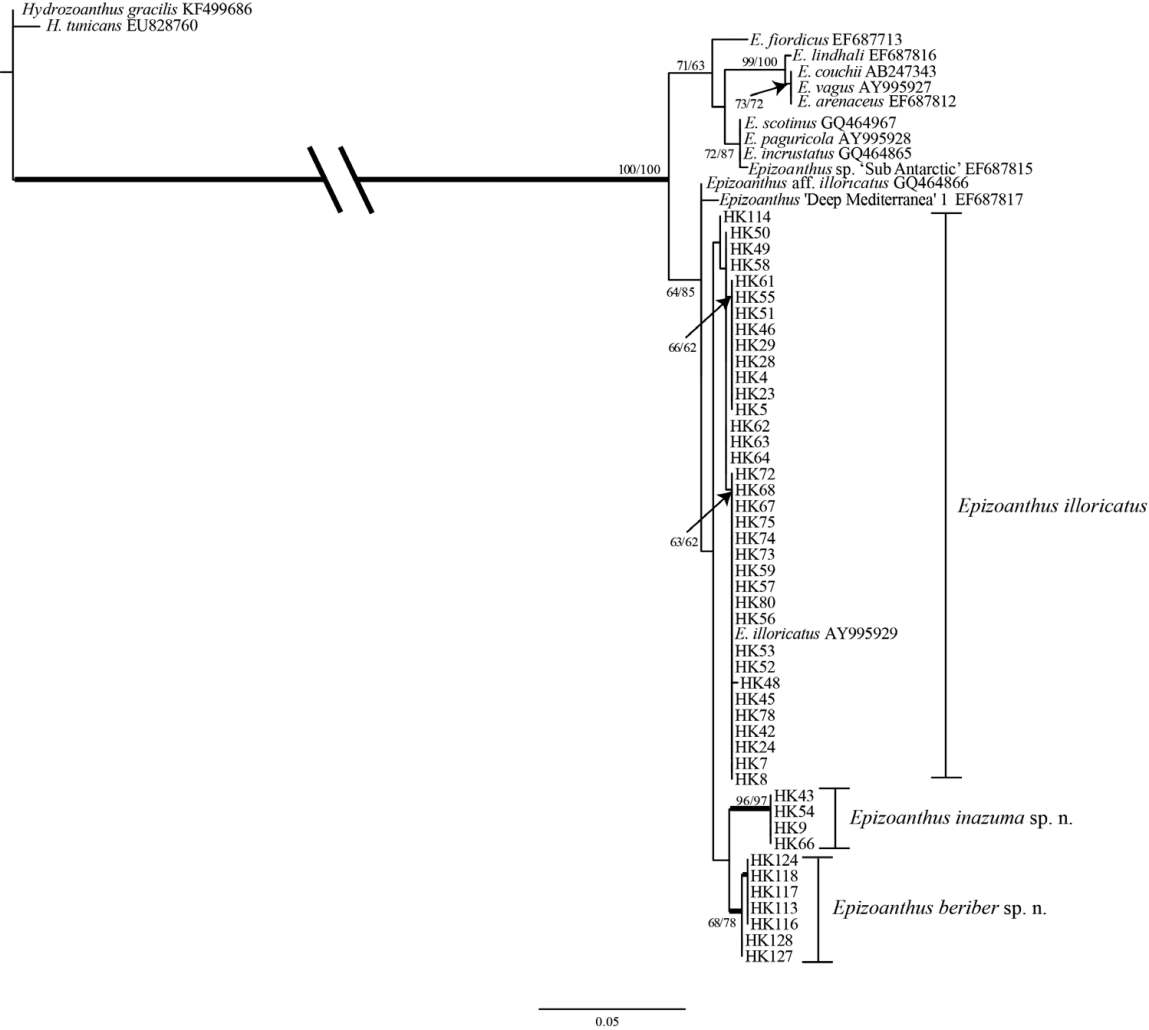
Maximum likelihood (ML) tree based on mitochondrial 16S ribosomal DNA sequence. Numbers on nodes represent ML and neighbor-joining (NJ) bootstrap values (> 50% are shown). Bold branches indicate high supports of Bayesian posterior probabilities (> 0.95). Sequences obtained from GenBank are shown with accession numbers.

**Figure 7.**
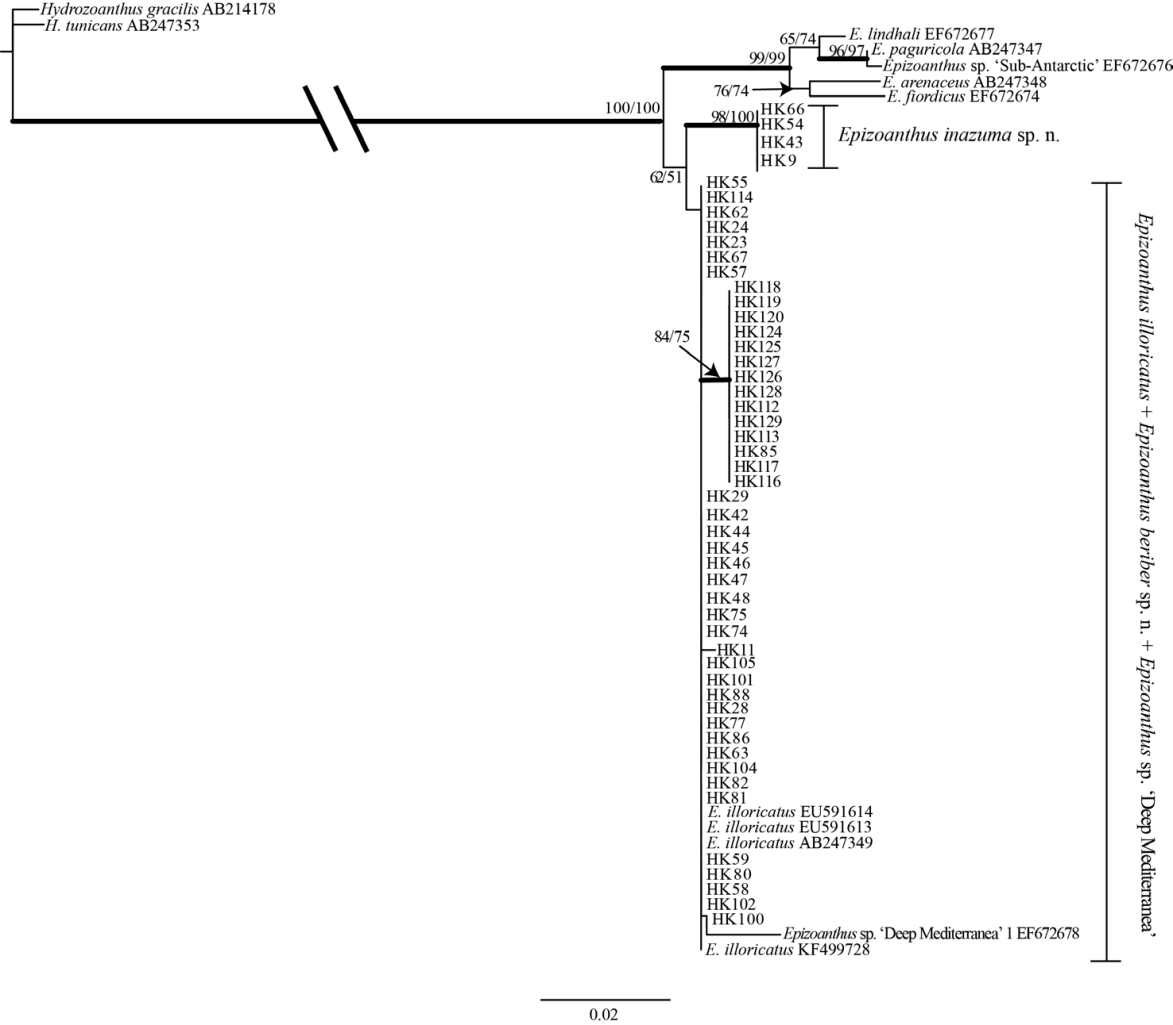
Maximum likelihood (ML) tree based on mitochondrial cytochrome oxidase subunit I sequence. Numbers on nodes represent ML and neighbor-joining (NJ) bootstrap values (> 50% are shown). Bold branches indicate high supports of Bayesian posterior probabilities (> 0.95). Sequences obtained from GenBank are shown with accession numbers.

Sequences from *Epizoanthus* spp. specimens attached to eunicid worm tubes formed a large monophyletic clade along with other *Epizoanthus* spp. in the phylogenetic tree of all three DNA markers (Figures 5–7). The phylogenetic trees’ topologies were very similar for all three DNA markers.

Although the morphological features of *Epizoanthus inazuma* sp. n. and *Epizoanthus beriber* sp. n. were generally very similar to those of *Epizoanthus illoricatus*, sequences were clearly separated into three monophyletic clades in the ITS-rDNA tree (Figure 5); all sequences of *Epizoanthus inazuma* sp. n. were contained in a monophyletic clade with very strong support (ML = 99%; NJ = 100%; Bayes = 1), and all sequences of *Epizoanthus beriber* sp. n. were also contained in another monophyletic clade with very strong support (ML = 100%; NJ = 100%; Bayes = 1). All sequences of *Epizoanthus illoricatus*, including previously reported sequences from GenBank, were contained in another monophyletic clade with strong support (ML = 95%; NJ = 97%; Bayes = 0.86).

The resulting trees from mt 16S-rDNA and COI sequences from specimens in this study also demonstrated that all three species were different (Figures 6–7, respectively); *Epizoanthus inazuma* sp. n. and *Epizoanthus beriber* sp. n. were each contained in monophyletic clades with moderate to strong support (COI: ML = 98%; NJ = 100%; Bayes = 1; and ML = 84%; NJ = 75%; Bayes = 0.97: mt 16S-rDNA: ML = 96%; NJ = 97%; Bayes = 0.99; and ML = 68; NJ = 78: Bayes = 0.98; respectively). There were 5-6 bp differences between *Epizoanthus beriber* sp. n. and *Epizoanthus illoricatus* in the each of the mt 16S-rDNA and COI regions.

Previously reported sequences of Epizoanthus aff. illoricatus (ITS-rDNA: GQ464895; mt 16S-rDNA: GQ464866) from Station M, Monterey Bay, California, USA were also contained within the clade of *Epizoanthus* spp. attached to eunicid worm tubes (Figures 5–6), although it is not clear which host this specimen was attached to (T. Swain, MorphBank collection number 477931 [MorphBank 2015]). In the ITS-rDNA tree, the sequence from this specimen was sister to a clade consisting of *Epizoanthus illoricatus* and *Epizoanthus beriber* sp. n. sequences with poor support (ML =< 50%; NJ = 55%; Bayes = 0.79) (Figure 5), and was sister to the large *Epizoanthus illoricatus*+*Epizoanthus inazuma* sp. n. +*Epizoanthus beriber* sp. n. clade in the mt 16S-rDNA tree (Figure 6). Previously reported sequences of *Epizoanthus* sp. ‘Deep Mediterranea’ 1 (mt 16S-rDNA: EF672678; COI: EF687817) were also contained in the clade of *Epizoanthus* spp. attached to eunicid worm tubes (Figures 6–7), although this specimen was apparently not associated with any living substrate (F. Sinniger, personal communication). This sequence was sister to a large, moderately well supported clade of *Epizoanthus illoricatus*, *Epizoanthus inazuma* sp. n., and *Epizoanthus beriber* sp. n. (ML = 64%; NJ = 85%; Bayes = 0.55) in the mt 16S-rDNA tree (Figure 6), and was contained in a clade with *Epizoanthus illoricatus* sequences in the COI tree (Figure 7).

## Discussion

Shallow *Epizoanthus* species associated with eunicid worm tubes are relatively common in the Pacific Ocean. However, until now there has been limited information about their diversity, and overall *Epizoanthus* species diversity is still relatively unknown and may be higher than has been originally thought (Reimer et al. 2010a). In this study two new species have been described, *Epizoanthus inazuma* sp. n. and *Epizoanthus beriber* sp. n. Based on these and previous findings (Sinniger et al. 2009; Reimer et al. 2010a), we believe there is a high potential of undescribed species being contained within already described *Epizoanthus* species. In this study, *Epizoanthus beriber* sp. n. was only found in caves. Similarly, two *Palythoa* species that live in similar habitats have recently been described from Okinawa (Irei et al. 2015), and an azooxanthellate scleractinian coral species was also discovered in similar habitats in various Indo-West Pacific localities, including Palau (Hoeksema 2012). Such findings indicate that there may be high potential of the existence of more undescribed species in underwater caves or other ‘cryptic’ environments associated with coral reefs, and continued investigations of such environments are needed.

### Distinguishing characters of different *Epizoanthus* species attached to eunicid worm tubes

*Epizoanthus illoricatus* has high levels of intraspecific morphological variation of some characters, such as external coloration, coenenchyme thickness, and polyp density (Figure 4a-c). Therefore, it may be easy to mistake different morphotypes as undescribed or potentially novel species by basing decisions only on morphological analyses, as has been suggested in other zoantharians (e.g. Burnett et al. 1997). In fact, although we collected some *Epizoanthus illoricatus* specimens that had poorly developed coenenchymes with a low density of polyps, other specimens had a thin, highly developed coenenchyme with a high density of polyps (Figure 4a–b), and these two different morphotypes were not consistently recovered in different phylogenetic clades. Thus, although these two morphotypes had recently been speculated to be different species (Reimer et al. 2014), this does not appear to be an accurate delineation of species. Additionally, *Epizoanthus inazuma* sp. n. looks very similar externally to *Epizoanthus illoricatus*. Thus, *Epizoanthus illoricatus* and *Epizoanthus inazuma* sp. n. may be easily mistaken for each other due to these similar morphological characteristics.

However, phylogenetic analyses clearly showed that *Epizoanthus illoricatus* and *Epizoanthus inazuma* sp. n. are clearly distinct and each is within a well-supported monophyly (Figures 5–7), with genetic distances of 0.4% to 1.3% in mt 16S-rDNA and COI regions separating them. Previous literature has shown such genetic distances to be in line with interspecific differences among other zoantharian congeners (Reimer et al. 2006, 2010a; Sinniger et al. 2008; Irei et al. 2015).

Between *Epizoanthus illoricatus* and *Epizoanthus inazuma* sp. n. we found notable differences in mesenteriel arrangements (Figure 2) and in cnidae; and in particular clear differences based on the presence/absence of large holotrichs (Table 1, Figure 3). Mesenterial arrangement is usually used as a taxonomic character to divide suborders, however our morphological analyses in this study indicate that mesenterial arrangement is an unreliable indicator of suborder. The results of our study also suggest that in some cases mesenterial arrangement may be useful for species-level identification when combined with molecular analyses and data from other morphological characteristics.

*Epizoanthus beriber* sp. n. can be easily distinguished from *Epizoanthus illoricatus* and *Epizoanthus inazuma* sp. n. by habitat and polyp coloration (Figure 4a–f). In general, species identification based on coloration has been thought of as not generally reliable for brachycneminic zoantharians as much intraspecific variation is present (Burnett et al. 1995, 1997; Reimer et al. 2004), while it has been supposed that coloration may be considered useful for identification of some macrocnemic zoantharians (Sinniger et al. 2009, 2010). Here, we consider coloration of polyps as a potentially useful taxonomic characteristic in these *Epizoanthus* species when utilized in combination with habitat data and molecular analyses.

### Relationship between *Epizoanthus* spp. and eunicid worm tubes

*Epizoanthus illoricatus*, *Epizoanthus inazuma* sp. n. and *Epizoanthus beriber* sp. n. are obligate epibionts on eunicid worms. Members of Eunicidae that host these *Epizoanthus* spp. make chitin-like zigzag tubes (Tischbierek 1930), and some colonies of *Epizoanthus illoricatus* completely covered this substrate. In this study, we observed no *Epizoanthus illoricatus* attached to tubes that did not have living eunicid worms inside. This means that *Epizoanthus illoricatus* apparently has some kind of association with living eunicid worms; commensalism, mutualism, or parasitism. To understand this relationship, observations of the survival rate of *Epizoanthus* colonies with or without eunicid worms in both controlled laboratory settings and in situ are necessary. *Epizoanthus illoricatus* and the two new species in this study do not produce tube-like structures such as a carcinoecium (Figure 4a–f), which is a corneous shell-like structure that has been observed in other *Epizoanthus* species’ associations (e.g. hermit crabs; Schejter and Mantelatto 2011). In addition, because there are few morphological differences despite clearly distinct phylogenetic signals between *Epizoanthus illoricatus* and *Epizoanthus inazuma* sp. n., it is possible that the substrate consisting of eunicid worm tubes may be made by different host taxa (genus/species). Further research using molecular and morphological analyses of not only *Epizoanthus* but also of the *Eunice* host species are needed to understand these relationships better.

## Supplementary Material

XML Treatment for Epizoanthus

XML Treatment for Epizoanthus inazuma

XML Treatment for Epizoanthus beriber

## Supplementary materials

Supplementary material 1 GenBank accession number, name and details of the sequences used in phylogenetic analyses of COI, mt 16S-rDNA and ITS-rDNA in this study Data type: GenBank accession numbers This dataset is made available under the Open Database License (http://opendatacommons.org/licenses/odbl/1.0/). The Open Database License (ODbL) is a license agreement intended to allow users to freely share, modify, and use this Dataset while maintaining this same freedom for others, provided that the original source and author(s) are credited.Hiroki Kise, James Davis Reimer

Supplementary material 2 List of examined specimens, and GenBank accession numbers Data type: GenBank accession numbers This dataset is made available under the Open Database License (http://opendatacommons.org/licenses/odbl/1.0/). The Open Database License (ODbL) is a license agreement intended to allow users to freely share, modify, and use this Dataset while maintaining this same freedom for others, provided that the original source and author(s) are credited.Hiroki Kise, James Davis Reimer
